# Tear Proteomics Approach to Monitoring Sjögren Syndrome or Dry Eye Disease

**DOI:** 10.3390/ijms20081932

**Published:** 2019-04-19

**Authors:** Ming-Tse Kuo, Po-Chiung Fang, Tsai-Ling Chao, Alexander Chen, Yu-Hsuan Lai, Yu-Ting Huang, Chia-Yi Tseng

**Affiliations:** 1 Department of Ophthalmology, Kaohsiung Chang Gung Memorial Hospital and Chang Gung University College of Medicine, Kaohsiung 83301, Taiwan; fangpc@cgmh.org.tw (P.-C.F.); a1050276@hotmail.com (A.C.); hurray@cgmh.org.tw (Y.-H.L.); r06100813@cgmh.org.tw (Y.-T.H.); ppp6692001@cgmh.org.tw (C.-Y.T.); 2 Department of Laboratory Medicine, Kaohsiung Chang Gung Memorial Hospital and Chang Gung University College of Medicine, Kaohsiung 83301, Taiwan; tsaeling@cgmh.org.tw

**Keywords:** dry eye disease, Sjögren syndrome, tears, homeostasis, biomarkers

## Abstract

Sjögren syndrome (SS) or dry eye disease (DED) is one of the most complicated ocular surface diseases. The goal of this study is to elucidate the relationship of the changes in clinical indices of tear film (TF) homeostasis with respect to tear components to allow for SS-DED monitoring and avoid stably controlled SS-DED patients from re-entering a vicious cycle. This prospective case-control study compared stable SS-DED patients with non-SS-DED control from several aspects, including clinical indices for TF homeostasis, 2 DED diagnostic biomarkers (MMP-9 and lactoferrin), and the proteome of flush tears. Compared with non-SS-DED controls, stably controlled SS-DED subjects had less tear secretion and higher ocular surface inflammation, a higher concentration ratio of tear MMP-9/lactoferrin, a more diverse tear proteome, and lower spectral intensities of lipocalin-1, lacritin, and prolactin-inducible protein among the abundant tear proteins. For stable SS-DED patients, the concentration ratio of tear MMP-9/lactoferrin and the corrected lipocalin-1 signal was positively correlated with ocular inflammation and TF stability, respectively. MMP-9 released from stressed ocular surface epithelium and lipocalin-1 secreted from the energetic lacrimal gland are two tear biomarkers responding well to TF homeostasis. The tear proteomics approach through flush tears is a promising method for monitoring SS-DED patients with a standardized sampling procedure and lactoferrin-corrected analysis.

## 1. Introduction

Dry eye disease (DED) is a distressing and even disabling disease that has been increasingly emphasized worldwide following its growing population [1]. The consensus of Tear Film and Ocular Surface Society in the Dry Eye Workshop II revised the definition of DED as a multifactorial disease of the ocular surface characterized by a loss of homeostasis of the tear film along with ocular symptoms of which tear film instability and hyperosmolarity, ocular surface inflammation and damage, and neurosensory abnormalities play etiological roles [2]. A loss of tear film homeostasis with ocular surface inflammation may be a response to changes in tear components. Therefore, it is highly valuable to identify the change of tear components by responding to the cyclical process of DED from breakdown of tear film homeostasis to ocular surface inflammation. Understanding the change of clinical indices of tear film homeostasis by the altering tear components will be helpful for eye care practitioners to monitor the treatment of DED and prevent stably controlled DED patients from re-entering the vicious cycle.

DED is more prevalent in patients with autoimmune diseases, which affect around 8% of the population, of whom 78% are women [3]. Sjögren syndrome (SS) is an autoimmune disorder characterized by exocrine gland dysfunction, which affects the lacrimal and salivary glands. SS-DED is predominantly associated with aqueous-deficiency. However, a higher rate of tear evaporation than that of the non-SS-DED has also been reported [4]. Among DED, SS-DED is the most severe group and is associated with the highest rates of complications, including filamentary keratitis, persistent epithelial erosion, corneal ulceration, melting, and even perforation. Therefore, exploring the association between tear film homeostasis and tear components of SS-DED is of utmost importance.

Despite the small volume for sampling, human tears offer several advantages for biochemical analysis and serves as a comprehensive biomolecule repertoire for biomarker discovery for diseases [5]. With the advancing genomics/transcriptomics/proteomics/metabolomics/lipidomics/glycomics technology, the biochemical changes in tears have been explored in several ocular diseases using ‘Omics’ approaches over 10 years. Omics aims at the collective characterization and quantification of pools of biochemical molecules that translate into the structure, function, and dynamics of an organism or organisms [6]. Accordingly, tear proteomics in this study include any set of characterization and quantification of pools of tear peptides/proteins that translate into the tear film, tear functional performance, and tear dynamics of human or other animals with tear secretion. We believe the tear proteomics approach will inspire researchers to evaluate the linkage between tear film homeostasis with potential tear biomarkers using the more comprehensive approach in the future.

Monitoring of tear biomarkers is essential for preventing recurrences of excruciating symptoms and severe ocular surface complications in SS-DED patients, but very few studies have emphasized the importance of DED monitoring [7,8]. Thus, the aim of this study was to elucidate the tear performance through a set of tear proteomic biomarkers in SS-DED patients under long-term and stable control. Moreover, the study also tried to clarify the associations between potential biomarkers and representative clinical tests in tear film homeostasis in these patients.

## 2. Results

### 2.1. Clinical Performance between Stable SS-DED and Non-SS-DED

A total of 20 female participants, including 10 stable SS-DED patients (five primary SS and five secondary SS, including three systemic lupus erythematous and two rheumatoid arthritis) and 10 non-SS-DED controls who met the inclusion and exclusion criteria were enrolled in this study (Table 1). Compared to the control subjects, stable SS-DED patients had a higher ocular surface disease index (OSDI) score, ocular surface staining, higher meiboscale, shorter tear break-up time (TBUT), lower tear meniscus height (TMH), and increased bulbar conjunctival redness. Although central TMH, the nasal bulbar redness score, and the mean redness score reached a significant difference, there was only a marginal trend toward significance in the OSDI score, TBUT, assessable time of TBUT, temporal TMH, temporal limbal bulbar redness score, and assessable area of bulbar redness. Accordingly, we selected a set of tests, including three significant indices (central TMH, nasal bulbar redness score, mean redness score) and two marginally significant indices (OSDI and NIKBUT first of TBUT) as clinical monitoring indices.

### 2.2. Representative Biochemical Markers of DED

**Figure 1:**
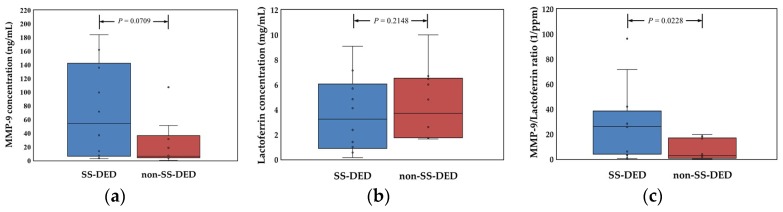
Comparison of the expression of two representative biochemical markers in dry eye patients with or without Sjögren syndrome. (**a**) Concentration of tear MMP-9. (**b**) Concentration of tear lactoferrin. (**c**) Concentration ratio of MMP-9 to lactoferrin of each tear sample. Statistical test by Mann Whitney U test, *p* < 0.05 was recognized as statistically significant.

Compared with the tears of non-SS-DED controls, SS-DED patients had a marginal trend toward significance in matrix metallopeptidase 9 (MMP-9) levels but not in lactoferrin concentration (Figure 1). However, the concentration ratio of MMP-9/lactoferrin was significantly higher in SS-DED patients.

### 2.3. Proteomic Spectra of Tears for SS-DED and Non-SS-DED

**Figure 2:**
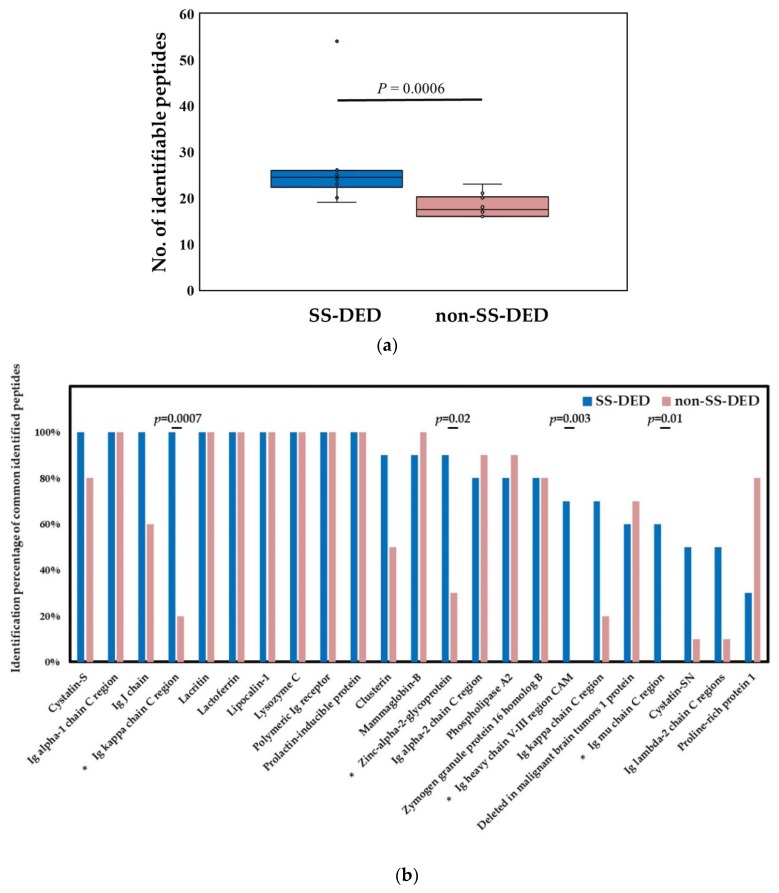
Tear proteome analyzed by the liquid chromatograph coupled tandem mass spectrometry in Sjögren syndrome and control subjects. (**a**) Comparison of the number of identified molecules. Statistical test by Mann Whitney U test, *p* < 0.05 was recognized as statistically significant. (**b**) Comparison of the isolation rate of common identified molecules. Statistical test by Fisher exact test, *p* < 0.05 was recognized as statistically significant (*).

SS-DED subjects had significantly more identified peptides or proteins in tears than non-SS-DED controls based on the liquid chromatograph coupled tandem mass spectrometry (LC-MS/MS) (Figure 2a). For commonly identified peptide/protein molecules, which were defined by an identification rate ≥50% for either group, there were four molecules more commonly detected in SS-DED subjects (Figure 2b), including immunoglobulin (Ig) kappa chain C region, zinc-alpha-2-glycoprotein, Ig heavy chain V-III region cellular adhesion molecule (CAM), and Ig mu chain C region, respectively. There were seven molecules with 100% presentation in both SS-DED and non-SS-DED subjects, including Ig alpha-1 chain C region, lacritin, lactoferrin, lipocalin-1, lysozyme C, polymeric Ig receptor, and a prolactin-inducible protein.

**Figure 3:**
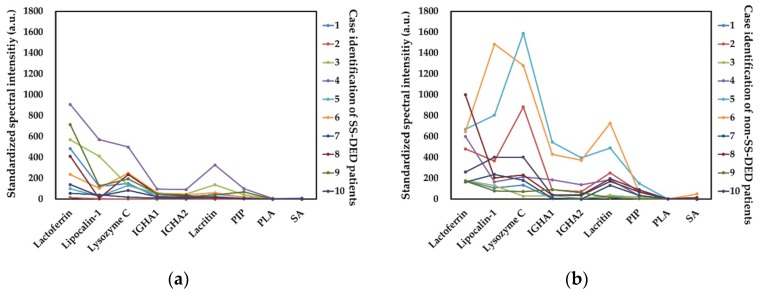
Spectral profile of lactoferrin-corrected standardized signals for nine selected molecules. (**a**) Tear spectra in Sjögren syndrome dry eye disease. (**b**) Tear spectra in non-Sjögren syndrome controls. IGHA1 = Immunoglobulin alpha-1 chain C region. IGHA2 = Immunoglobulin alpha-2 chain C region. PIP = Prolactin-inducible protein. PLA = Phospholipase A2. SA = serum albumin. a.u. = arbitrary unit.

**Figure 4:**
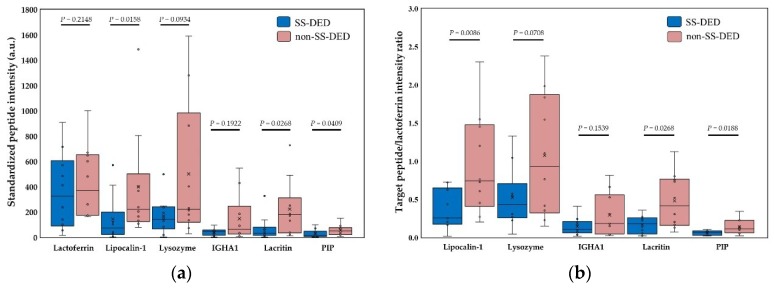
Comparison of corrected tear spectral intensities for Sjögren syndrome patients and non-Sjögren syndrome controls. (**a**) Standardized spectral intensity corrected by lactoferrin concentration for six always identified molecules. (**b**) The spectral intensity ratio of a specific molecule to lactoferrin. IGHA1 = Immunoglobulin alpha-1 chain C region. PIP = Prolactin-inducible protein. a.u.= arbitrary unit. Statistical test by Mann Whitney U test, *p* < 0.05 was recognized as statistically significant.

Nine molecules, including six always presented molecules and three arbitrarily selected molecules, were adopted for mass spectral intensity analysis (Figure 3). The signal intensity was generally higher in non-SS-DED controls, but both SS-DED and non-SS-DED subjects had similar spectral profiles constructed by these selected molecules. Contrary to the identified molecules in the mass spectrum, SS-DED patients had lower mass spectral intensities of tear molecules. Among the six present molecules, the lactoferrin-corrected mass spectral intensities of lipocalin-1, lacritin, and prolactin-inducible protein were significantly lower in SS-DED patients (Figure 4a). In addition, the spectral intensity ratio of the selected molecule to lactoferrin also showed similar results (Figure 4b).

### 2.4. Association between Clinical Performance and Biochemical Markers in SS-DED

**Figure 5:**
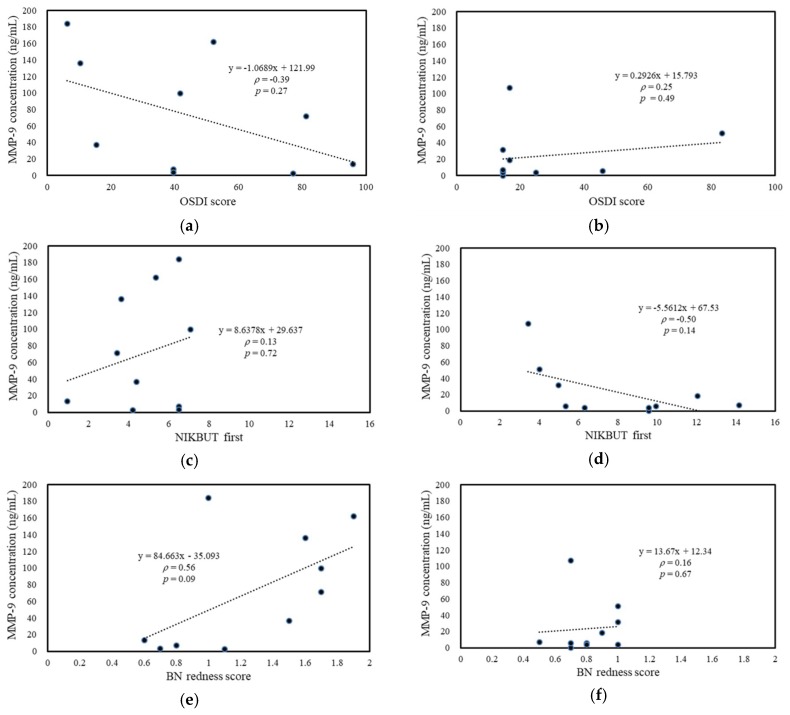

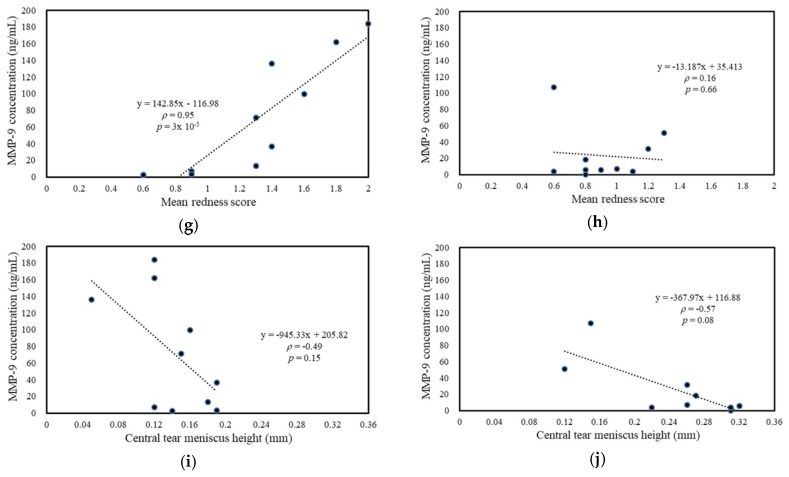
Association between ocular surface homeostatic markers and tear MMP-9 concentration. (**a**,**c**,**e**,**g**,**i**) SS-DED patients. (**b**,**d**,**f**,**h**,**j**) Non-SS-DED controls. BN = bulbar nasal.

**Figure 6:**
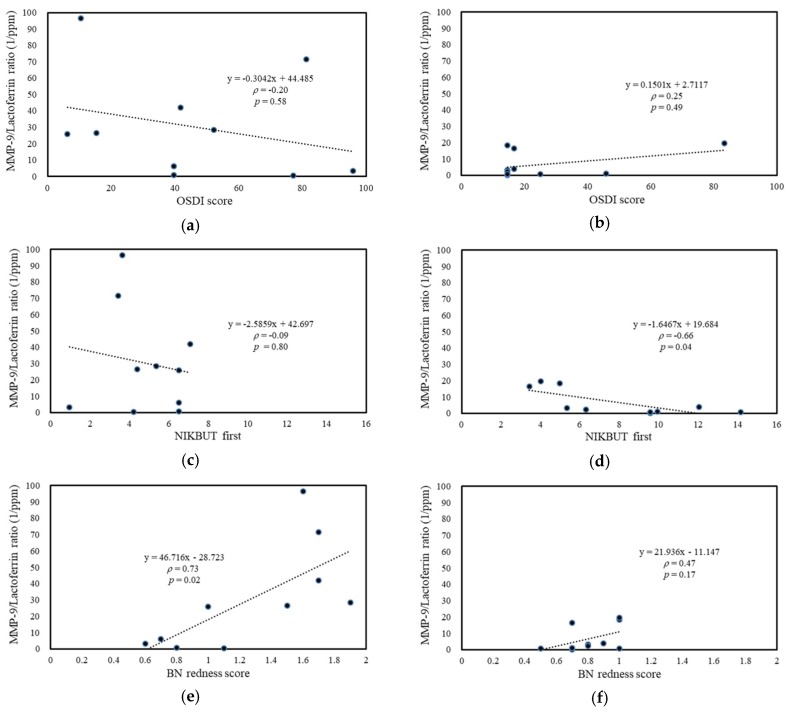

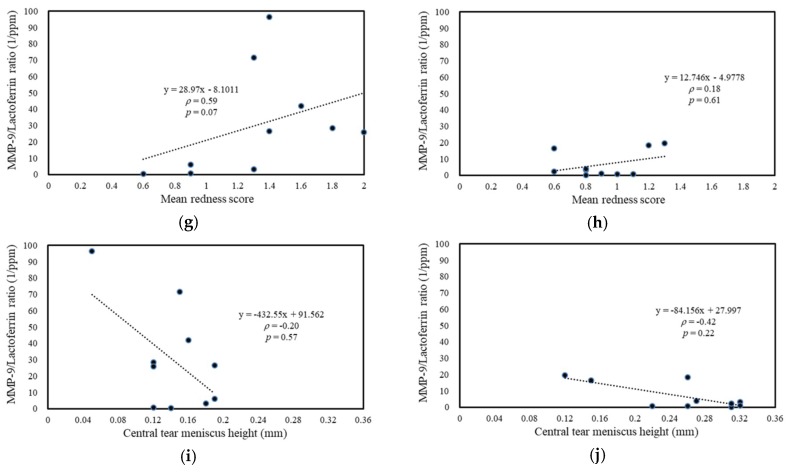
Association between ocular surface homeostatic markers and tear MMP-9/Lactoferrin concentration ratio. (**a**,**c**,**e**,**g**,**i**) SS-DED patients. (**b**,**d**,**f**,**h**,**j**) non-SS-DED controls. BN = bulbar nasal.

For SS-DED patients, the tear MMP-9 level was positively correlated with bulbar redness, including mean redness score (with significance), and nasal bulbar redness (with a marginal trend to significance) (Figure 5g,e). For non-SS-DED controls, tear MMP-9 was negatively corrected with central tear meniscus height (with a marginal trend to significance) (Figure 5j). Tear MMP-9/lactoferrin concentration ratio in SS-DED patients was also positively correlated with bulbar redness, including nasal bulbar redness (with significance) and mean bulbar redness (with a marginal trend to difference) (Figure 6e,g), while this ratio in non-SS-DED controls was negatively and significantly correlated with TBUT (Figure 6d). For both SS-DED and non-SS-DED subjects, OSDI was not correlated with a tear MMP-9 level or an MMP-9/lactoferrin ratio. The associations between clinical tests and the two potential biochemical markers were summarized in Table 2. 

### 2.5. Association between Clinical Performance and Proteomic Spectra in SS-DED

**Figure 7:**
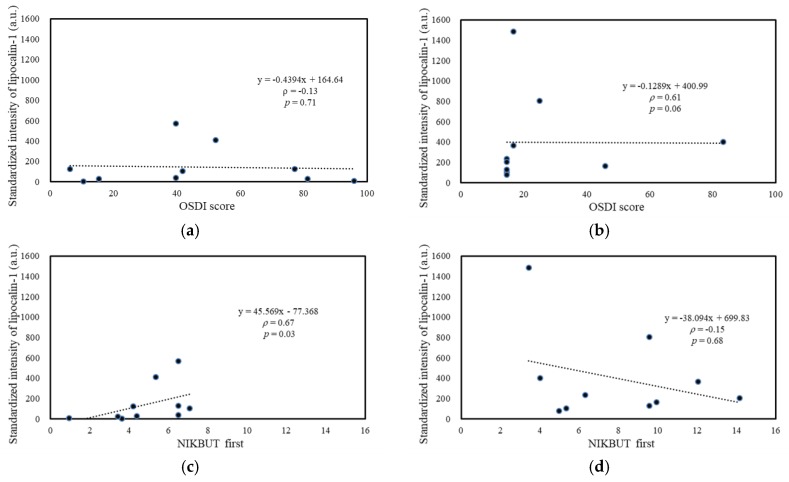

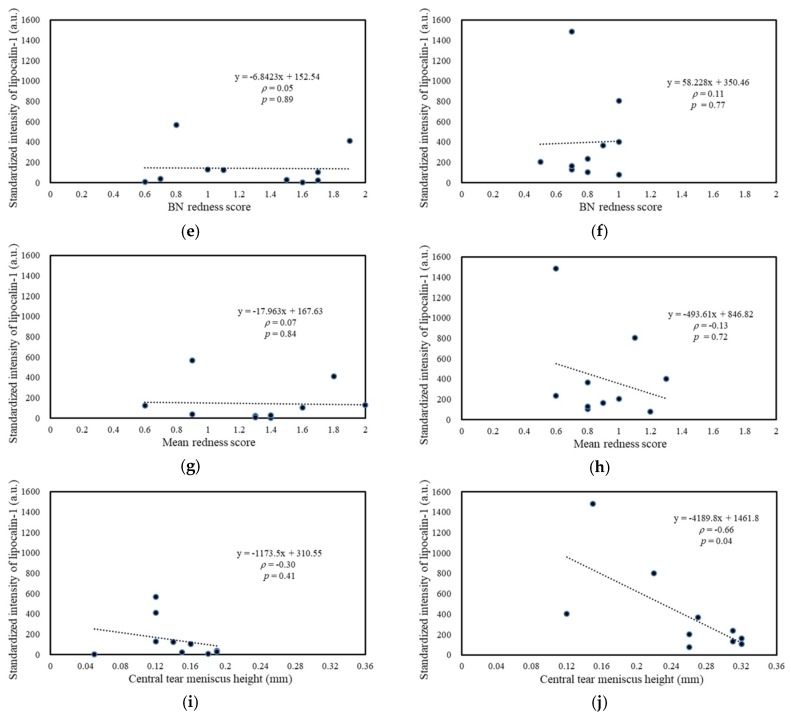
Association between ocular surface homeostatic markers and standardized signal intensity of tear Lipocalin-1. (**a**,**c**,**e**,**g**,**i**) SS-DED patients (**b**,**d**,**f**,**h**,**j**). Non-SS-DED controls. BN = bulbar nasal.

High correlations were observed among the standardized spectral intensities of lipocalin-1, lacritin, and prolactin-inducible protein (data not shown) because these abundant tear proteins were majorly produced by the lacrimal gland. Lipocalin-1, the tear protein with the most significant spectral difference between SS-DED and non-SS-DED, was selected as the spectral tear proteomic marker for the following analysis. Standardized intensity of lipocalin-1 was positively and significantly correlated with TBUT (Figure 7c) in SS-DED patients, but negatively correlated with central TMH (with significance, Figure 7j) and an OSDI score (with a marginal trend to significance, Figure 7b) in non-SS-DED controls. However, no significant correlation between the lipocalin-1 signal and bulbar redness was observed for both SS-DED and non-SS-DED subjects.

## 3. Discussion

We hypothesized that stably controlled SS-DED patients had different tear performance from non-SS-DED subjects without any treatment. However, the tear film homeostasis of SS-DED patients under effective treatment may not be easily clarified by a single test. Thus, we proposed a novel tear proteomics concept aimed at combining potential tear biomarkers and proteome for monitoring SS-DED during stable care. In this study, we found stable SS-DED patients still had significantly less tear secretion (central TMH) and higher inflammation (redness scores) in the clinical performance, while SS-DED patients only had a marginal trend toward significance in the increase of dry eye symptoms (OSDI) and the decrease of dynamic tear film stability (TBUT) (Table 1). In addition, two point-of-care tear biomarkers, MMP-9 and lactoferrin levels, might not be enough to serve as independent tests for monitoring stable SS-DED patients (Figure 1). However, the concentration ratio of tear MMP-9/lactoferrin was significantly and positively correlated with ocular inflammation (nasal bulbar redness score) in these SS-DED subjects (Figure 6). Moreover, stable SS-DED subjects had more diverse spectra and different identification rates of tear components than a non-SS-DED control (Figure 2), but had lower signal intensities in the commonly abundant proteins by LC-MS/MS (Figure 3). Furthermore, the corrected spectral intensities of lipocalin-1, lacritin, and prolactin-inducible protein in stable SS-DED subjects were significantly lower than those in the non-SS-DED subjects (Figure 4). For these SS-DED patients, a stronger signal of lipocalin-1 represented more stable tear film stability (higher TBUT) but was not associated with lower ocular inflammation (redness score) (Figure 7).

Serum immunoglobulin free light chains were previously reported as sensitive biomarkers for monitoring disease activity in SS [9]. However, there were no testing standards for monitoring SS-DED tear film homeostasis [8]. Versura et al. found the concentrations of tear proteins, especially lactoferrin and lipocalin-1, provided an excellent diagnostic performance compared with the traditional ocular clinical tests for early diagnosis of SS [10]. In vitro diagnostics based on tears was a non-invasive or mini-invasive strategy for diagnosing DED. Tear MMP-9 [11,12,13] and lactoferrin [14] levels had been developed as point-of-care diagnostic tools for DED, but the two tear biomarkers had not been considered as monitoring biomarkers for SS-DED. In our study, an insignificantly elevated tear MMP-9 level may imply some stable SS-DED subjects had less stressed epithelial cells that produced inflammation-facilitating cytokines or a diluted effect via flush tear sampling. Stable SS-DED patients had similar tear lactoferrin levels to non-SS-DED controls. This result might suggest the surviving lacrimal gland cells were able to compensate with respect to the level of lactoferrin production or even over-producing lactoferrin given the consideration of the diluted effect of flush tear sampling. The tear concentration ratio of MMP-9/Lactoferrin may be recognized as a normalized index to minimize the dilution effect of flush tear sampling. In addition, MMP-9 and lactoferrin, respectively, reflect the status of injuries of ocular surface cells and the function of lacrimal gland secretion. MMP-9 increase and lactoferrin decrease will follow the worsening status of DED. The significantly higher tear concentration ratio of MMP-9/Lactoferrin in stably controlled SS-DED patients possibly implied that this index is a more objective and sensitive biomarker for monitoring purposes.

The tear proteomics approach was limited as a laboratory tool and varied by different MS-based proteomic strategies for a small volume of the tear sample. SS-DED patients particularly have a small volume of tears. Many previous studies adopted the pooling and eluted tear sample for discovering the subtle changes of vital proteomes in comparative study groups. Some researchers used label-free protein quantification [15,16,17,18], while others used isotopic labeling protein quantification [19,20], which is inherently more complex and often prohibits the detection of the low-abundance tear proteins [18]. In this study, we adopted a standardized normal saline flush procedure to collect tear samples efficiently when obtaining a personal tear mass spectrum of each subject. In addition, we analyzed the label-free corrected intensity of mass spectra of commonly identified molecules and directly explored the association of standardized spectral intensities with clinical TF homeostasis markers. Although some proteins with a very low concentration (trace tear proteins, such as MMP-9) might not be detectable with this approach, the proteomes composed of common molecules with higher concentration (abundant tear proteins, such as lactoferrin) can be clarified for each subject. By avoiding complex functional annotation, our approach directly clarified the relationship between abundant tear proteins and clinical performance.

Li et al. investigated the tear proteome of SS-DED subjects and used a pooled tear sample from a non-anesthetized Schirmer test strip for 2D LC-MS/MS analysis [15]. This study analyzed the normalized spectral counting and reported 10 downregulated proteins in SS-DED subjects, which are highly compatible with the results based on standardized spectral intensity in our SS-DED subjects. We found lipocalin-1, lacritin, and prolactin-inducible protein were still significantly downregulated in stably controlled SS-DED patients. Li et al. concluded that the tear characterization of SS-DED patients is an altered proteomic profile with dysregulated expression of proteins associated with several important cellular processes including inflammation, immunity, and oxidative stress. Our study supported these findings and further elucidated some downregulated abundant proteins that were correlated with dynamic tear film stability in SS-DED subjects. However, Aqrawi et al. analyzed the CD9^+^ extracellular vesicles in pooled tears by size exclusion chromatography column and found SS-DED patients had upregulation of neutrophil-gelatinase lipocalin and overexpression of proteins involved in TNF-α signaling and B cell survival [21]. This result implied some tear biomarkers may have been obtained from the trace proteins, which were released from the cellular response to ocular surface inflammation.

Although the flush method is a verified tear sampling procedure [22], it carried the concerns of diluting the basal tears, activating the corneal nerves, and inducing reflex tears to change the tear protein profile. However, no approach in tear sampling can guarantee a reflex tears-free sample. Moreover, some SS-DED subjects had nearly no tears for collection. Therefore, the standardized flush tear collection method with basal or reflex tear collection methods was adopted in this study. No apparent reflex tearing was identified during collection and each subject did not report discomfort during tear sampling. In addition, the corrected indices, such as the tear MMP-9/lactoferrin ratio and lactoferrin-corrected spectral intensity, minimized the dilution effect of the flush tear sampling.

In this study, the sample size was determined by the OSDI difference between active SS-DED patients and normal controls. Some indices of clinical tests and a candidate tear biomarker only reached a marginal difference or correlation. Therefore, the monitoring potential of some tear biomarkers needs further investigation in the future. Nevertheless, we still found that the tear MMP-9 and lipocalin-1 were respectively linked with ocular surface inflammation and dynamic tear film stability in stably controlled SS-DED patients.

## 4. Materials and Methods

### 4.1. Participants

This prospective case-control study, which was part of an investigation of ocular adnexal microorganisms, enrolled female DED patients under long-term follow-up and stable control with tear lubricants at the corneal department of Kaohsiung Chang Gung Memorial Hospital (CGMH) between 1 November 2018 and 31 January 2019. Informed consent was obtained from all subjects, and all procedures adhered to the Declaration of Helsinki and the ARVO statement on human subjects. Institutional Review Board/Ethics Committee approval was obtained from the Committee of Medical Ethics and Human Experiments of CGMH, Taiwan (201600708B0, 28 July 2016).

Subjects were classified as stably controlled SS-DED and non-SS-DED groups. The stably controlled SS-DED subjects previously met the DED diagnosis (met OSDI > 13, and at least one positive for TBUT < 10 s or Oxford staining score > 1) and were previously diagnosed as SS by rheumatologists by respective classification criteria [23,24,25], including primary SS and secondary SS who had another well-defined systemic autoimmune disease. At enrollment, these patients had tolerable or no ocular symptoms under current treatment regimen, no filamentary keratitis, and no increased ocular punctate erosions over 3 months. Non-SS-DED subjects did not have SS, which included normal subjects (met OSDI ≤ 13, TBUT ≥ 10 s, and Oxford staining score ≤ 1) and DED subjects (met OSDI > 13, and at least one positive for TBUT < 10 s or Oxford staining score > 1) without the need for eyedrop treatment. Those who were less than 20 years old had acute ocular inflammation or glaucoma, underwent ocular or eyelid surgery within 6 months, had diabetes mellitus, or were pregnant were excluded. In each group, subject enrollment was closed after the group met the sample size requirement. The right eye of each subject was used for assessing tear film homeostasis, tear biochemical markers, and tear proteome.

### 4.2. Assessment Protocol and Tear Sample Collection

The evaluation procedures for each subject were performed in the same order. First, each subject completed a questionnaire, OSDI, for assessing the subjective severity of dry eye. Second, the right eye of each subject was assessed for ocular surface homeostasis by masked examiners according to the following order: TMH, ocular surface redness scan (R-scan), and TBUT, which was followed by the assessment of meibography of lower eyelid. Third, tears were collected at least 30 min after the above examination. Each subject was asked to lie down on an operation table in a supine position for tear collection without topical anesthesia. Tears were sampled according to a standardized eye-flush procedure [22] by instilling a single 60 μL drop of non-preserved, unit dose normal saline to the corneal center of the right eye with gentle eyelid support by the left forefinger and thumb of the collector. The subject was instructed to keep the eye open, move the eye around, then gaze to the left, and slightly tilt the head to the right about 10−15°. Under an operating microscope, 20 μL tear fluid sample was gently and quickly obtained by a steady collection of tears pooling in the superior fornix near the lateral canthus with a 1 to 20 μL automated pipetting system (Pipetty, Icomes Lab Co., Ltd., Iwate, Japan) held by the right hand. The collected tears were immediately spinned down by centrifugation at 6000× *g* in a microcentrifuge for 10 min at 4 °C and the supernatant liquid was stored at −20 °C. Lastly, each subject was examined by a slit lamp bio-microscope for assessment of ocular surface staining based on an Oxford scheme [26].

### 4.3. Determining the Subjective Severity of Dry Eye by the OSDI Questionnaire

There are a total of 12 questions in the OSDI questionnaire [27]. Each question is scored from 0 to 4 by frequency (none of the time to all of the time), and total score is calculated by the sum of scores for all questions answered and divided by the total number of answered questions. The OSDI is to inquire the frequency of DED-related discomfort events within one week, including five items about the common ocular symptoms, four items about vision-related functional impairment, and three items about the uncomfortable feeling induced by certain environments. The OSDI is assessed on a scale of 0 to 100, with higher scores representing greater disability. Each patient responded to the questions after being instructed by a masked examiner. After completion of the questionnaire, the score was calculated and recorded for each subject.

### 4.4. Selected Ocular Surface Homeostasis Markers of DED

There were three basic classes of ocular surface homeostasis markers adopted in this study for clinical performance measurement, including tear secretion indices, ocular surface inflammatory indices, and dynamic tear film stability indices. With a tear film analyzer (Keratograph^®^ 5M, Oculus, GmbH, Wetzlar, Germany), each patient was examined noninvasively and all the above indices were objectively obtained.

#### 4.4.1. Determination of Tear Secretion

Each subject underwent imaging with the tear film analyzer with illumination by means of four infrared diodes with an 880-nm wavelength [28]. These diodes were mounted on the Placido ring keratograph in a horizontal orientation and arranged in two pairs located one above the other. The white ring illumination used for corneal topography was deactivated, which ensured a dark background for the assessment. The measurements were performed three times for three seconds after each blink for each subject. Because the height of the tear meniscus varies along the curve of the lower eyelid, the intersected point of the lower lid margin curve and the elongated line connecting the central cornea with 4-o’clock, 6-o’clock, and 8-o’clock limbus was used for measuring tear meniscus height of nasal meniscus, central meniscus, and temporal meniscus, respectively. These tear meniscus heights were then measured with an integrated ruler.

#### 4.4.2. Assessment of Ocular Surface Inflammation

Each subject underwent the R-scan after changing the light source to a white ring illumination by the same tear film analyzer [29,30]. Each patient was instructed to stare straight ahead and focus on the fixation mark inside the camera after blinking to allow the 22 mire Placido ring system to be reflected in the entire corneal area. The R-scan detects blood vessels in the conjunctiva and evaluates the degree of redness. The built-in software automatically separates the regions of interest as the bulbar nasal (BN) region, the bulbar temporal (BT) region, the limbal nasal (LN) region, and the limbal temporal (LT) region, and returns redness using a clinical grading scale of 0.0–4.0 in 0.1 steps. Six indices were obtained within 10 s, including the BN score, the BT score, the LN score, the LT score, the mean redness score, and the assessable area.

#### 4.4.3. Evaluation of Dynamic Tear Film Stability

The assessment of non-invasive TBUT was used to elucidate the dynamic tear film stability for each subject. All subjects underwent imaging with the tear film analyzer under 880-nm ring illumination to prevent glare during the examination [31,32]. Subjects were instructed to fixate centrally, blink twice in a natural way, and forcefully suppress their blink for as long as they could. The mire rings projected on the corneal surface were captured by videokeratoscopy. The corneal topographic recording started automatically right after the second blink and lasted maximum 23 s. TBUT was measured noninvasively as the time between the last complete blink and the first distortion of Placido rings projected onto the surface of the cornea, which the device detects automatically. The tear film analyzer generates three indices for TBUT: (1) the NIKBUT-first records the time at which the first perturbation in the reflected Placido disk pattern occurs, (2) the NIKBUT-avg calculates the average time of all detected perturbation and is related to localized TBUTs, and (3) the assessable time shows the time from the beginning of the recording to the last complete blink. It is related to tolerability to suppress the blink.

### 4.5. Selected Biochemical Markers of DED

The tear sample was thawed on ice for detecting the concentrations of tear components, measuring the total protein, and analyzing the tear proteome within four weeks after collection. MMP-9 and lactoferrin, which was previously used to diagnose the DED, were selected as two representative biochemical markers to assess the DED-induced change of tear components for these subjects. MMP-9 production increases in response to hyperosmolar conditions of ocular surface, contributes to corneal barrier disruption, and rises with increasing levels of DED severity [13]. Lactoferrin secreted from a major lacrimal gland binds to iron in tear fluid, exerts anti-microbial, antitumor, and immunomodulatory properties, and maintains homeostasis of ocular surface health [14]. Lactoferrin levels in tear fluid are reduced in SS and non-SS dry eye patients. The concentration of tear components was recalculated by a 2.5× dilution factor estimated by a prior test comparing the total protein levels of basal tears collected by capillary tube and eye-flush tears collected by automated micropipette for the same subject.

#### 4.5.1. Measurement of the MMP-9 Concentration of Tears

The concentrations of MMP-9 of all tear samples were analyzed by using a sandwich enzyme-linked immunosorbent assay (ELISA) kit for human MMP-9 (Arigo, Hsinchu, Taiwan). The assay was performed in duplicate and carried out at 37 °C in accordance with the instructions of the manufacturer. Four-μL tear sample was further diluted with the ELISA buffer of the manufacturer to a final volume of 100 μL. The measurement was performed at a wavelength of 450 nm and analyzed using a Microplate Reader (Thermo Scientific Multiskan FC with internal ELISA Software, Vantaa, Finland).

#### 4.5.2. Measurement of the Lactoferrin Concentration of Tears

The concentrations of lactoferrin of all tear samples were analyzed by using a quantitative sandwich ELISA for human lactoferrin (Abnova, Taipei, Taiwan). The assay was performed at room temperature in duplicate, according to the instructions of the manufacturer. One μL tear sample was diluted with the ELISA buffer, according to the manufacturer’s dilution guideline. The measurement was performed at a wavelength of 450 nm and analyzed using the same Microplate Reader (Thermo Scientific Multiskan FC with internal ELISA Software, Vantaa, Finland).

### 4.6. Evaluation of Tear Proteome

The total protein of each tear sample was quantified by using the Bradford protein-binding assay (Bio-Rad Protein Assay, Bio-Rad Laboratories Taiwan Ltd., Taipei, Taiwan). The volume of the tear sample was adjusted to obtain the same 5 μg total protein in each sample for proteomic analysis.

#### 4.6.1. In-Solution Digestion of Proteins

The sample was mixed with 10 μL of 100 mM ammonium bicarbonate, 5.0 μL of 0.2 g/L trypsin, and 27.5 μL ddH_2_O. The mixer was then incubated for 6 h at 37 °C. Subsequently, 0.5 μL of 0.5 M dithiotreitol solution was added, which was followed by incubation for 30 min at 56 °C. Reduced cysteine residues were alkylated by adding 1.5 μL of 0.5 M iodoacetamide solution and incubation proceeded for 30 min at room temperature in the dark. Then, 0.5 μL of 0.5 M dithiotreitol solution was added, which was followed by incubation for 30 min at 37 °C. The sample was added with 5.0 μL of 0.2 g/L trypsin in a 1:30 (trypsin/protein) mass ratio and diluted with 42.5 μL ddH_2_O to a final volume of 100 μL. After incubation overnight at 37 °C, the sample was heated for 5 min at 100 °C. Each sample was then frozen and dried at −80 °C. The sample was restored with 25 μL of 0.1% trifluoroacetic acid. Subsequently, 3 μL of the restored sample was analyzed by the liquid chromatograph coupled tandem mass spectrometry (LC-MS/MS).

#### 4.6.2. LC-MS/MS

An HCT Ultra ETDII Ion-trap Mass Spectrometer (Bruker Daltonics) interfaced with an UltiMate 3000 nano high performance liquid chromatography system (Dionex) with a 15 cm × 75 μm C18 column was used per the Jian study [33]. In brief, the peptides of the sample were eluted using an acetonitrile gradient at a flow rate of 0.3 mL/min. The spectra for the eluted fractions were acquired as successive sets of scan modes. The MS scan obtained the intensity of ions in the range of 200 to 2000 *m*/*z*, and a specific ion was selected for a tandem MS/MS scan. The centroid MS/MS data of enzyme-digested fragments were obtained using HyStar 3.2, Bio-Tools, and WarpLC software (Bruker Daltonics). Subsequently, the data were submitted to a bioinformatics search program (MASCOT) for searching Swiss-Prot databases based on Homo Sapiens with the following settings: a mass tolerance of 0.3 Da for precursor and fragment ions, one missed cleavage acceptable for trypsin digestion, carbamidomethyl cysteine residues as fixed modifications, and oxidized methionine residues for an optional modification.

### 4.7. Sample Size Determination

The sample size was calculated by a free online calculator (http://www.sample-size.net) developed and maintained by the Clinical and Translational Sciences Institute (CTSI) of UCSF. According to the OSDI results of SS and non-SS DED patients reported in the Versura study [34], we estimated the sample size by adopting the significance level (α) as 0.05, the desired power (1-β) as 0.8, the effect size of 15, and the standard deviation of the population of 10. Accordingly, the estimated sample size was at least nine subjects in each group, and 10 subjects in each group were determined to be the sample size of this study.

### 4.8. Data Analysis

For comparing the spectral intensity of peptides among different samples, the lactoferrin spectral intensity was used as a reference, according to the lactoferrin concentration determined by ELISA. The 1 mg/mL lactoferrin was assigned as 100 equivalent spectral intensity (arbitrary unit), and the equivalent spectral intensity of lactoferrin was calculated for each subject. The standardized intensity of a specific molecule was then calculated by the absolute spectral amplitude of the specific molecule divided by the absolute spectral amplitude of lactoferrin from the same mass spectrum and multiplied by the equivalent spectral intensity of lactoferrin of the same subject.

The statistical analysis was performed by using the Analysis ToolPak of Microsoft Excel (2016) and the Social Science Statistics (http://www.socscistatistics.com/tests/signedranks/Default2.aspx), which is free online software. All clinical and tear proteomic indices was tested by the Mann–Whitney U test for SS and non-SS DED patients, except the Fisher exact test was used to compare the isolation rate of common identified molecules for the 2 groups. A general linear regression model and Spearman’s rank correlation coefficient was used to explore the association among indices for SS and non-SS DED subjects. A *p*-value of <0.05 was considered statistically significant.

## 5. Conclusions

Even under effective treatment, stably controlled SS-DED patients still have a different tear biochemical profile from non-SS-DED subjects. Tear MMP-9 and lipocalin-1 were identified as potential biomarkers in monitoring SS-DED patients because of a distinct expression from non-SS-DED subjects and a significant correlation with clinical performance. The tear proteomics approach through flush tears using a standardized sampling procedure and corrected analysis shows great promise in monitoring SS-DED patients. We look forward to this approach as being one useful way to implement precision medicine in patients with ocular surface disease in the future.

## Figures and Tables

**Table 1:** Demographic data of dry eye patients with or without Sjögren syndrome.

Clinical Parameters	SS-DED (*n* = 10)	Non-SS-DED (*n* = 10)	*p* Value
Age (years)	48.8 ± 14.2	47.6 ± 9.5	0.67
OSDI	45.9 ± 30.9	24.2 ± 23.8	0.08
Oxford staining score	1.3 ± 0.5	0.8 ± 0.6	0.13
Meiboscale	1.8 ± 0.6	1.7 ± 0.5	0.69
Tear break-up time (s)			
NIKBUT first	4.9 ± 1.9	7.9 ± 3.6	0.06
NIKBUT avg	8.7 ± 5.0	11.5 ± 4.5	0.09
Assessable time	12.8 ± 7.6	17.2 ± 7.3	0.06
Tear meniscus height (mm)			
Nasal meniscus	0.33 ± 0.22	0.36 ± 0.12	0.18
Central meniscus	0.14 ± 0.04	0.25 ± 0.07	0.002 *
Temporal meniscus	0.25 ± 0.11	0.35 ± 0.15	0.07
Bulbar redness			
BN score	1.3 ± 0.5	0.8 ± 0.2	0.02 *
BT score	1.2 ± 0.5	1.0 ± 0.4	0.13
LN score	0.9 ± 0.5	0.7 ± 0.3	0.16
LT score	0.9 ± 0.5	0.6 ± 0.4	0.09
Mean redness score	1.3 ± 0.4	0.9 ± 0.2	0.01 *
Assessable area	12.8 ± 7.6	17.2 ± 7.3	0.06

All variants were shown in mean ± SD. Statistical test by Mann-Whitney U test, *p* < 0.05 was recognized as statistically significant (*). Abbreviation: SS, Sjögren syndrome, DED, dry eye disease, OSDI, ocular surface disease index, NIKBUT first, non-invasive keratographic first break-up time, NIKBUT avg, non-invasive keratographic average break-up time, BN, bulbar nasal, BT, bulbar temporal, LN, limbal nasal, and LT, limbal temporal.

**Table 2:** The associations between clinical tests and two potential biochemical markers.

Clinical Tests		SS-DED		Non-SS-DE	
		MMP-9	MMP-9/LF	MMP-9	MMP-9/LF
OSDI score	β	−1.07	−0.30	0.29	0.15
	ρ	−0.39	−0.20	0.25	0.25
	*p* value	0.27	0.58	0.49	0.49
NIKBUT first (s)	β	8.64	−2.59	−5.56	−1.65
	ρ	0.13	−0.09	−0.50	−0.66
	*p* value	0.72	0.80	0.14	0.04 *
Central TMH (mm)	β	−945.3	−432.6	−368.0	−84.2
	ρ	−0.49	−0.20	−0.57	−0.42
	*p* value	0.15	0.57	**0.08**	0.22
BN redness score	β	84.7	46.7	13.7	21.9
	ρ	0.56	0.73	0.16	0.47
	*p* value	**0.09**	0.02 *	0.67	0.17
Mean redness score	β	142.8	29.0	−13.2	12.7
	ρ	0.95	0.59	0.16	0.18
	*p* value	3 × 10^−5^ *	0.07	0.66	0.61

β is the slope of equation of simple liner regression between a clinical test and a biochemical marker. ρ is the Spearman’s correlation coefficient. *p* value < 0.05 was recognized as a statistically significant correlation (*) and *p* value < 0.1 was highlighted in bold. Abbreviation: SS, Sjögren syndrome. DED, dry eye disease. OSDI, ocular surface disease index. NIKBUT first, non-invasive keratographic first break-up time. TMH, tear meniscus height. BN, bulbar nasal.

## Abbreviations

DED	dry eye disease
SS	Sjögren syndrome
OSDI	ocular surface disease index
TBUT	tear film break-up time
NIKBUT	non-invasive keratographic break-up time
TMH	tear meniscus height
R-scan	ocular surface redness scan
BN	bulbar nasal
BT	bulbar temporal
LN	limbal nasal
NT	limbal temporal
LC-MS/MS	liquid chromatography coupled tandem mass spectrometry
